# Diseases of Women

**Published:** 1898-07

**Authors:** 


### Books and Magazines.

Diseases of Women. A Clinical Guide to their Diagnosis and Treatment, by G. Ernest Herman, M. B, Lond., F. R. C. P. Obstetric Physician to and Lecturer on Midwifery at the London Hospital; Consulting Physician-Accoucheur to the Tower Hamlets Dispensary; Examiner in Midwifery to the Universities of London and Oxford; Late President of the Obstetrical Society of London and of the Hunterian Society; Formerly Physician to the General Lying-in Hospital and to the Eastern District of the Royal Maternity Charity, and Examiner in Midwifery to the Royal College of Surgeons. One Volume of 886 pages, octavo, profusely illustrated. Extra Muslin, $5.00 net, Leather, $5.75 net. William Wood & Co., Publishers, New York City. 1898.

This work, if viewed as it should be, as an account of the author's personal experience, is valuable. If viewed as a comprehensive and thorough text book of the subject it is not to be recommended. The author's intention, however, as stated by himself in the preface is only to write what he *knows*. It is not to be expected that any one man has the knowledge of this subject sufficient to write a text book. The author is evidently a very learned man and has a large clinical experience to draw upon. His style is clear and graphic though somewhat quaint. One phrase which is upon almost every page is, "I know not." It is refreshing to know that if your author is not certain of the truth of the statement he makes he will candidly say so; thus assuring you that he has a positive knowledge of the truth of all unqualified assertion of fact. One is tempted, however, to say that if he would discard such antiquated instruments as Ferguson's speculum, which he prefers, he might have known more. Some criticism might be made of his asepsis, or rather; lack of it. For instance his teachings for curetting the uterus consists in a vaginal douche of a $\frac{1}{2000}$ sublimate solution and immersing the *instruments* in the same, and nothing said of their being previously rendered surgically clean, or of any attention to cleanliness of the operator's hands. These are minor defects, however, and as an offset to them it can be truthfully said that there is a deep vein of rich clinical knowledge running through the work which is well worth the publisher's price to any physician. J.

